# Projections of Lung Cancer Incidence by 2035 in 40 Countries Worldwide: Population-Based Study

**DOI:** 10.2196/43651

**Published:** 2023-02-17

**Authors:** Ganfeng Luo, Yanting Zhang, Jaione Etxeberria, Melina Arnold, Xiuyu Cai, Yuantao Hao, Huachun Zou

**Affiliations:** 1 School of Public Health (Shenzhen), Sun Yat-sen University Shenzhen China; 2 Department of Medical Statistics, School of Public Health, Sun Yat-sen University Guangzhou China; 3 Department of Epidemiology and Health Statistics, School of Public Health, Guangdong Medical University Dongguan China; 4 Department of Statistics, Computer Science and Mathematics, Public University of Navarre Navarre Spain; 5 Institute for Advanced Materials and Mathematics (INAMAT2) Public University of Navarre Navarre Spain; 6 Cancer Surveillance Branch, International Agency for Research on Cancer Lyon France; 7 Department of VIP Inpatient, Sun Yat-sen University Cancer Center, State Key Laboratory of Oncology in South China, Collaborative Innovation Center for Cancer Medicine Guangzhou China; 8 Peking University Center for Public Health and Epidemic Preparedness & Response, Peking University Beijing China; 9 Kirby Institute, University of New South Wales Sydney Australia

**Keywords:** lung cancer, incidence, projections, temporal trends, worldwide

## Abstract

**Background:**

The global burden of lung cancer (LC) is increasing. Quantitative projections of the future LC burden in different world regions could help optimize the allocation of resources and provide a benchmark for evaluating LC prevention and control interventions.

**Objective:**

We aimed to predict the future incidence of LC in 40 countries by 2035, with an emphasis on country- and sex-specific disparities.

**Methods:**

Data on LC incidence from 1978 to 2012 were extracted from 126 cancer registries of 40 countries in *Cancer Incidence in Five Continents Volumes V-XI* and used for the projection. Age-standardized incidence rates (ASRs) per 100,000 person-years and the number of incident cases were predicted through 2035, using the NORDPRED age-period-cohort model.

**Results:**

Global ASRs of the 40 studied countries were predicted to decrease by 23% (8.2/35.8) among males, from 35.8 per 100,000 person-years in 2010 to 27.6 in 2035, and increase by 2% (0.3/16.8) among females, from 16.8 in 2010 to 17.1 in 2035. The ASRs of LC among females are projected to continue increasing dramatically in most countries by 2035, with peaks after the 2020s in most European, Eastern Asian, and Oceanian countries, whereas the ASRs among males will continue to decline in almost all countries. The ASRs among females are predicted to almost reach those among males in Ireland, Norway, the United Kingdom, the Netherlands, Canada, the United States, and New Zealand in 2025 and in Slovenia in 2035 and even surpass those among males in Denmark in 2020 and in Brazil and Colombia in 2025. In 2035, the highest ASRs are projected to occur among males in Belarus (49.3) and among females in Denmark (36.8). The number of new cases in 40 countries is predicted to increase by 65.32% (858,000/1,314,000), from 1.31 million in 2010 to 2.17 million in 2035. China will have the largest number of new cases.

**Conclusions:**

LC incidence is expected to continue to increase through 2035 in most countries, making LC a major public health challenge worldwide. The ongoing transition in the epidemiology of LC highlights the need for resource redistribution and improved LC control measures to reduce future LC burden worldwide.

## Introduction

### Background

Globally, lung cancer (LC) represents the first and third most commonly diagnosed cancer among males and females in 2020, respectively [1]. In 2020, an estimated 2.2 million new LC cases occurred worldwide, representing 14.3% (1.4 million) and 8.4% (0.8 million) of all new cancer diagnoses among males and females, respectively [1]. LC incidence rates have been steadily decreasing among males in most high-income countries (eg, European and Oceanian countries) in recent decades, whereas they have been rapidly increasing among females [2-4].

### Research Significance

However, the global LC burden continues to increase owing to population growth, population aging, and changes in the prevalence of risk factors (eg, smoking and air pollution), which will further exert tremendous strain on populations and health systems worldwide [2-4]. Quantitative projections of the future LC burden in different world regions, in terms of expected number of new cases and incidence rates in both absolute size and trends, can help optimize the allocation of resources for screening, diagnosis, and therapy and provide a benchmark for evaluating LC prevention and control interventions worldwide, with the aim of reducing the LC burden and promoting health equity [5].

### Objective

Historical LC incidence trends have been comprehensively evaluated worldwide [2-4,6]. However, to our knowledge, no previous study has predicted the future burden of LC incidence on a global scale. We aimed to quantify the future LC incidence in 40 countries by 2035 using long-standing and high-quality population-based data, with an emphasis on country- and sex-specific disparities.

## Methods

### Data Sources

Data on LC incidence (International Classification of Diseases 10th Revision C33-34) stratified by a 5-year period of diagnosis (1978-1982, ... , 2008-2012), sex, and 5-year age group were extracted from national and regional population-based cancer registries available in *Cancer Incidence in Five Continents* volumes V to XI [7,8]. The specific requirements for the inclusion of a registry were at least 15 consecutive years of data and compilation in the latest volume (XI) of the *Cancer Incidence in Five Continents* series [2,9,10]. These criteria are indicative of each registry’s data quality over time, given that the editorial process involves a detailed assessment of the comparability, completeness, and validity of the incidence data [2,9,10]. A total of 126 cancer registries from 40 countries in 10 world regions (Northern Europe, Western Europe, Southern Europe, Central and Eastern Europe, North America, Central and South America, Eastern Asia, Southeastern Asia, Southwestern Asia, and Oceania) were included. Of these, 19 countries contributed national data for the analysis (Denmark, Estonia, Iceland, Ireland, Lithuania, Norway, Austria, the Netherlands, Croatia, Cyprus, Malta, Slovenia, Belarus, Bulgaria, the Czech Republic, Slovakia, Costa Rica, Israel, and New Zealand). For the remaining 21 countries (the United Kingdom, France, Germany, Switzerland, Italy, Spain, Poland, Canada, the United States, Brazil, Chile, Colombia, Ecuador, China, Japan, Republic of Korea, Philippines, Thailand, India, Turkey, and Australia), data from multiple regional registries in a given country were aggregated to obtain a proxy for the national incidence. Population predictions (medium-fertility variant) were obtained from the United Nations World Population Prospects 2022 Revision up to 2035 by country, year, sex, and age [11].

### Statistical Analysis

Incidence rates per 5-year period of diagnosis and 5-year age group by sex were calculated based on the corresponding incident cases and population data from population-based cancer registries. Age-standardized incidence rates (ASRs) per 100,000 person-years were calculated based on the world standard population [12]. The number of cases by age group at the national scale were estimated by applying regional age-specific incidence rates to the national populations [9,10]. Lifetime cumulative risks were calculated for those aged 0 to 74 years and are expressed as percentages, which can be interpreted as the probability that an individual will develop LC before the age of 75 years in the absence of competing causes of death [13].

In this study, the well-known NORDPRED age-period-cohort model [9,10,14,15] was used to predict the number of new LC cases and incidence rates from 2013 to 2017 to 2033 to 2037 by country, sex, and age. Let us assume that, conditional on rates, the number of incident LC cases follows a Poisson distribution:



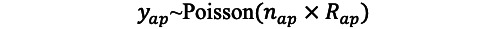



Where *y_ap_* and *n_ap_* represent the number of incident cases and person-years at risk, respectively, in the age group *a*=0−4, ... , 85+ and in the period *P*=1978-1982, ... , 2008-2012.

Then, the NORDPRED age-period-cohort model using a power-5 link function is defined as follows:



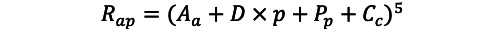



In this expression, *A_a_* is the age component of age group *a*; *D* is the common linear drift parameter of both calendar period *p* and birth cohort *c*; is the nonlinear period component of period ; and is the nonlinear cohort component of cohort *c*. Birth cohort *c* was extracted by subtracting the midpoint of the 5-year age group *a* from the corresponding midpoint of the 5-year calendar period P [2]. The 3 to 7 recent 5-year observed periods (depending on data availability) were extrapolated using a power-5 link function to level off growth, with a projection of drift parameter *D* that was attenuated by 25%, 50%, 75%, and 75% in the second, third, fourth, and fifth prediction periods, respectively [14]. Furthermore, we assessed the prediction performance of the NORDPRED age-period-cohort model, with detailed information and the corresponding findings presented in Multimedia Appendix 1 [15-20].

The projected number of new cases in 2035 were calculated by applying the predicted age-specific rates for 2033 to 2037 to the United Nations national population forecasts for 2035 for each country [14]. Mean annual differences in the number of predicted cases in 2035 (the midpoint of calendar period 2033-2037) relative to 2010 (the midpoint of calendar period 2008-2012) are partitioned into changes in risk (rates) and changes in demographics (population size and age structure) [15]; the detailed information is presented in Multimedia Appendix 1. The number of predicted cases and ASR in 2035 are presented and compared with those in 2010. A percentage change of ≥5% was defined as an increase or decrease; otherwise, the number and rates were considered stable [21]. Throughout the paper, the number of cases has been rounded to avoid spurious precision. In some cases, this creates small discrepancies with the displayed totals and percentages, which are based on the data before rounding.

Data management and modeling analyses were performed using R software (version 4.0.2; R Foundation for Statistical Computing) [22] and the NORDPRED package.

### Ethical Considerations

This study does not involve human participants and animal subjects. Ethics approval was not required for this study as the study used existing nonidentifiable data that were aggregated and population level.

## Results

### Changes in LC Incidence Rates: 2010 to 2035

**Figure 1:**
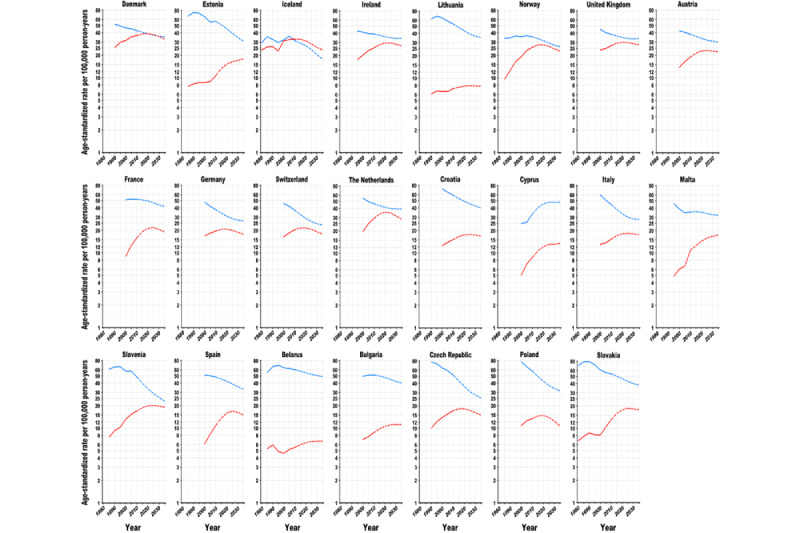
Trends in age-standardized (world) incidence rates of lung cancer in European countries, 1980 to 2035: observed rates (solid line) and projected rates (dashed line). The blue line stands for males, and the red line stands for females. Age-standardized (world) rates per 100,000 person-years.

**Figure 2:**
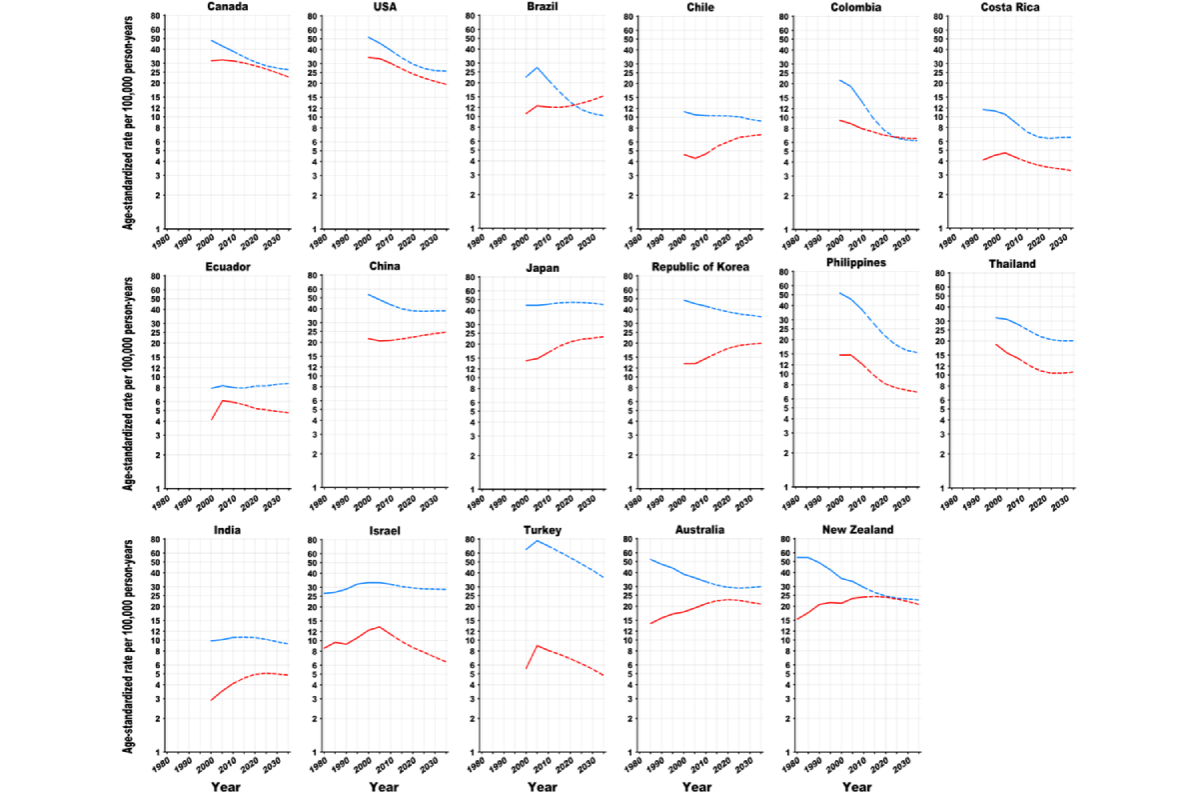
Trends in age-standardized (world) incidence rates of lung cancer in American, Asian, and Oceanian countries, 1980 to 2035: observed rates (solid line) and projected rates (dashed line). The blue line stands for males, and the red line stands for females. Age-standardized (world) rates per 100,000 person-years.

**Figure 3:**
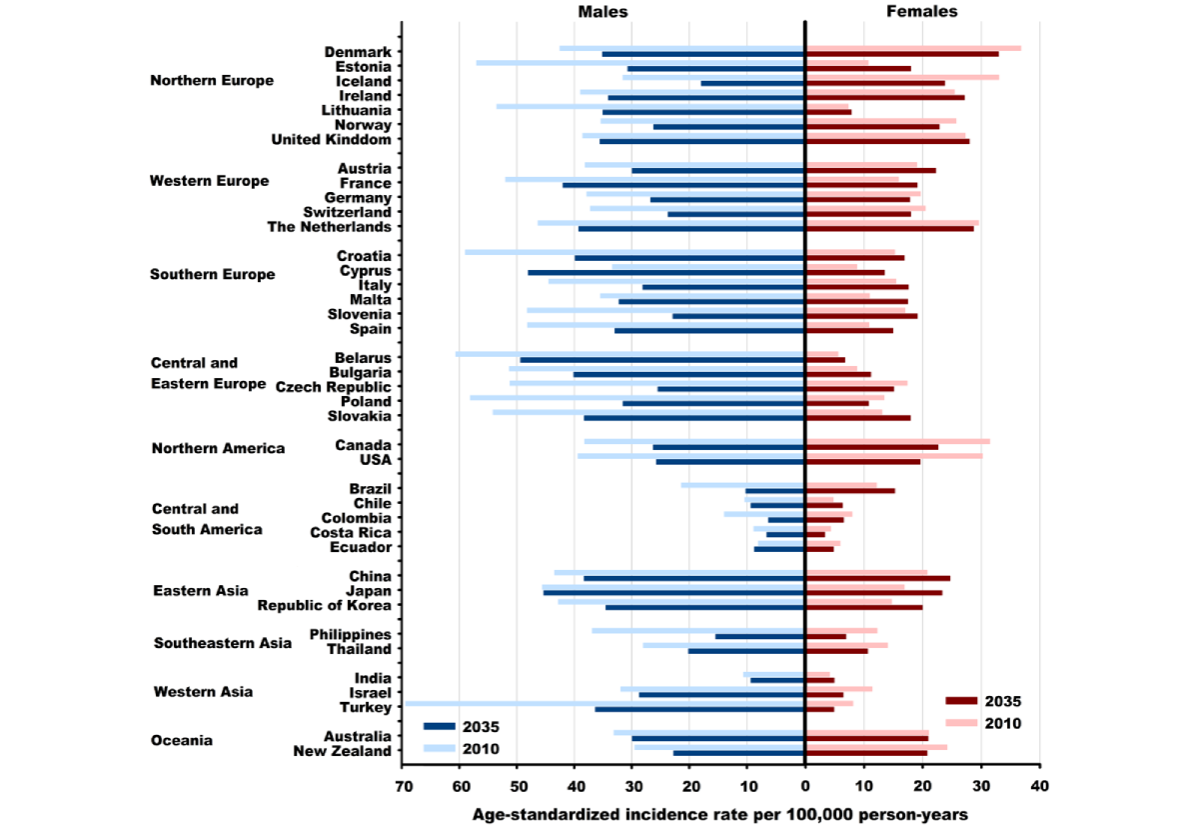
Lung cancer age-standardized incidence rates in 2010 and projected rates in 2035 in 40 countries. Rates for 2010 and 2035 represent average rates for the 5-year period centered on the respective year. Blue color stands for males, and red color stands for females. Age-standardized (world) rates per 100,000 person-years.

Global ASRs of the 40 studied countries are predicted to decrease by 23% (8.2/35.8) among males, from 35.8 per 100,000 person-years in 2010 to 27.6 in 2035, and increase by 2% (0.3/16.8) among females, from 16.8 in 2010 to 17.1 in 2035. Among the 40 studied countries, the ASRs are predicted to decline among males in 37 countries and among females in 17 countries between 2010 and 2035 (Figures 1-3; Multimedia Appendices 2 and 3). The largest decreases in ASRs are projected in the Philippines (−21.5/36.9, −58%; ASR 36.9 per 100,000 person-years in 2010 vs 15.4 in 2035), Colombia (−7.7/13.9, −55%; 13.9 vs 6.2), and Slovenia (−25.3/48.2, −53%; 48.2 vs 22.9) among males and in the Philippines (−5.3/12.2, −44%; 12.2 vs 6.9), Israel (−5/11.4, −43%; 11.4 vs 6.4), and Turkey (−3.3/8.1, −40%; 8.1 vs 4.8) among females. For each region, the largest reductions in ASRs are projected in Estonia (−26.3/57, −46%; ASR 57.0 per 100,000 person-years in 2010 vs 30.7 in 2035) for males and in Iceland (−9.2/33.0, −28%; 33.0 vs 23.8) for females in Northern Europe; in Switzerland for both sexes (−13.5/37.2, −36%; 37.2 vs 23.7 for males; −2.4/20.4, −12%; 20.4 vs 18.0 for females) in Western Europe; in Slovenia (−25.3/48.2, −53%; 48.2 vs 22.9) for males in Southern Europe; in the Czech Republic (−25.7/51.2, −50%; 51.2 vs 25.5) for males and in Poland (−2.7/13.4, −20%; 13.4 vs 10.7) for females in Central and Eastern Europe; in the United States for both males (−13.7/39.4, −35% 39.4 vs 25.7) and females (−10.6/30.2, −35%; 30.2 vs 19.6) in North America; in Colombia (−7.7/13.9, −55%; 13.9 vs 6.2) for males and in Costa Rica (−1.0/4.3, −24%; 4.3 vs 3.3) for females in Central and South America; in the Republic of Korea (−8.0/42.8, −19%; 42.8 vs 34.8) for males in Eastern Asia; in the Philippines for both sexes (−21.5/36.9, −58%; 36.9 vs 15.4 for males; −5.3/12.2, −44%; 12.2 vs 6.9 for females) in Southeastern Asia; in Turkey (−33.0/69.3, −48%; 69.3 vs 36.3) for males and in Israel (−5.0/11.4, −43%; 11.4 vs 6.4) for females in Southwestern Asia; and in New Zealand for both sexes (−6.8/29.5, −23%; 29.5 vs 22.7 for males; −3.4/24.2, −14%; 24.2 vs 20.8 for females) in Oceania.

However, the ASRs will increase in 2 countries for males and in 20 countries for females from 2010 to 2035.

Among males, percentage increases in ASRs are predicted in Cyprus (14.6/33.4, 44%; ASR 33.4 per 100,000 person-years in 2010 vs 48.0 in 2035) and Ecuador (0.7/8.0, 9%; 8.0 vs 8.7). among females, the largest increases in ASRs were predicted in Estonia (7.3/10.7, 68%; ASR 10.7 per 100,000 person-years in 2010 vs 18.0 in 2035), Malta (6.5/10.9, 60%; 10.9 vs 17.4), and Cyprus (4.8/8.7, 54%; 8.7 vs 13.5). As for each region, the steepest increases in ASRs are predicted in Estonia (7.3/10.7, 68%; ASR 10.7 per 100,000 person-years in 2010 vs 18.0 in 2035) in Northern Europe; France (3.3/15.8, 20%; 15.8 vs 19.1) in Western Europe; Malta (6.5/10.9, 60%; 10.9 vs 17.4) in Southern Europe; Slovakia (4.8/13.1, 37%; 13.1 vs 17.9) in Central and Eastern Europe; Chile (1.6/4.7, 33%; 4.7 vs 6.3) in Central and South America; Japan (6.5/16.8, 38%; 16.8 vs 23.3) in Eastern Asia; and India (0.8/4.1, 19%; 4.1 vs 4.9) in Southwestern Asia. The ASRs among females are predicted to peak after the 2020s in 27 countries (most European, Eastern Asian, and Oceanian countries), whereas those among males have already peaked in the historical period in almost all countries (except for Cyprus, Ecuador, and Japan).

**Figure 4:**
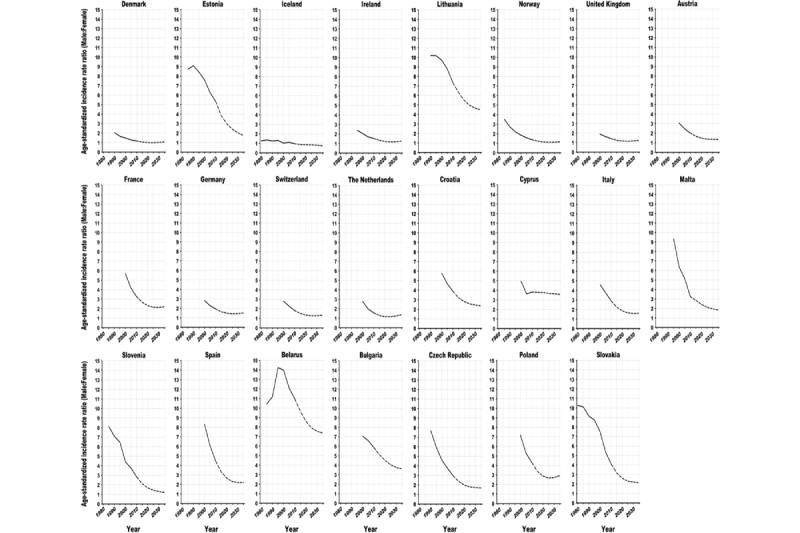
Trends in the male to female age-standardized (world) incidence rate ratios of lung cancer in European countries, 1980 to 2035: observed rate ratios (solid line) and projected rate ratios (dashed line).

**Figure 5:**
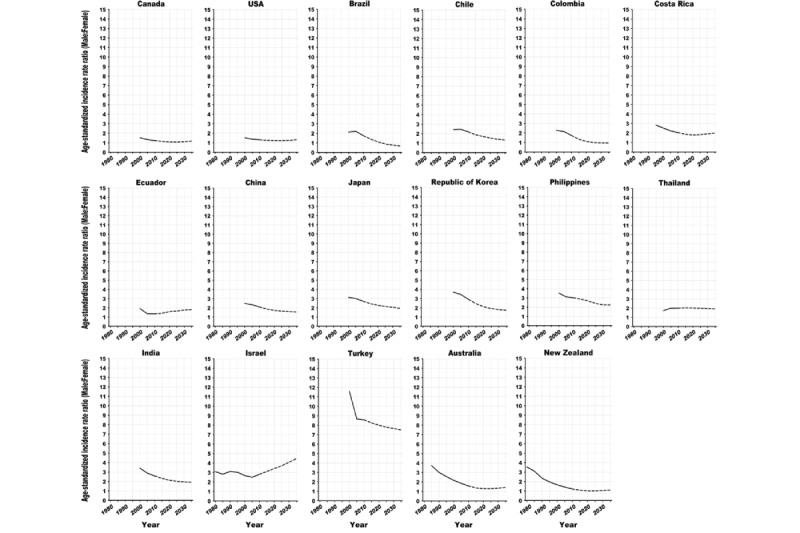
Trends in the male to female age-standardized (world) incidence rate ratios of lung cancer in American, Asian, and Oceanian countries, 1980 to 2035: observed rate ratios (solid line) and projected rate ratios (dashed line).

Although the ASRs among males have been historically higher than those among females in all countries (except for Iceland), the sex gaps are gradually narrowing (Figures 4 and 5). The male to female ASR ratios are projected to decline in 33 countries from 2010 to 2035. The largest reductions in male to female ratios are predicted for Estonia (−3.6/5.3, −68%; sex ratio 5.3 in 2010 vs 1.7 in 2035), Brazil (−1.1/1.8, −62%; 1.8 vs 0.7), Slovenia (−1.6/2.8, −58%; 2.8 vs 1.2), Spain (−2.2/4.4, −50%; 4.4 vs 2.2), and Slovakia (−2.0/4.1, 48%; 4.1 vs 2.1). ASRs among females are predicted to almost reach those among males in Ireland, Norway, the United Kingdom, the Netherlands, Canada, the United States, and New Zealand in 2025; in Slovenia in 2035; and even surpass those among males in Denmark in 2020 and in Brazil and Colombia in 2025.

In 2035, LC incidence is projected to vary 8-fold among males and 10-fold among females across countries (Figure 3; Multimedia Appendices 2 and 3). The highest ASRs were projected in Belarus (49.3 per 100,000 person-years), Cyprus (48.0), Japan (45.3), France (42.0), and Bulgaria (40.1) among males and in Denmark (32.9), the Netherlands (28.7), the United Kingdom (28.0), Ireland (27.1), and China (24.7) among females. The male to female ratio is projected to vary 11-fold across countries, ranging from 0.7 in Brazil to 7.5 in Turkey.

### Changes in the Number of New LC Cases: 2010 to 2035

Overall, the number of new cases in the 40 included countries is predicted to increase by 50.19% (429,000/854,000), from approximately 0.85 million in 2010 to approximately 1.28 in 2035, among males and is set to nearly double, from approximately 0.46 million to approximately 0.89, among females (Multimedia Appendices 2 and 3). When stratified by geographic region, the most rapid increases were predicted in Eastern Asia (342,000/433,000, 79.00% for males and 303,000/216,000, 140.05% for females).

**Figure 6:**
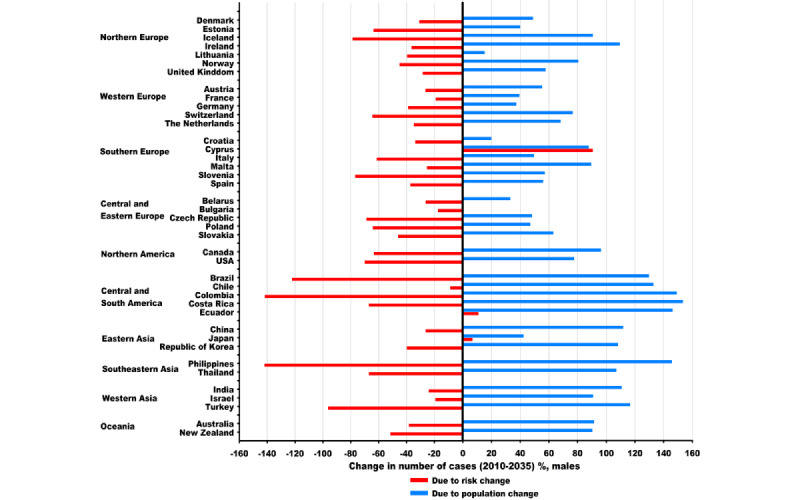
Projected changes in the number of new lung cancer cases between 2010 and 2035 in 40 countries among males, partitioned into population and risk change. Numbers for 2010 and 2035 represent average numbers for the 5-year period centered on the respective year. Due to population change: projected change in the number of cases due to population growth and aging. Due to risk change: projected change in the number of cases due to changes in incidence rates. Blue color stands for population change, and red color stands for risk change.

**Figure 7:**
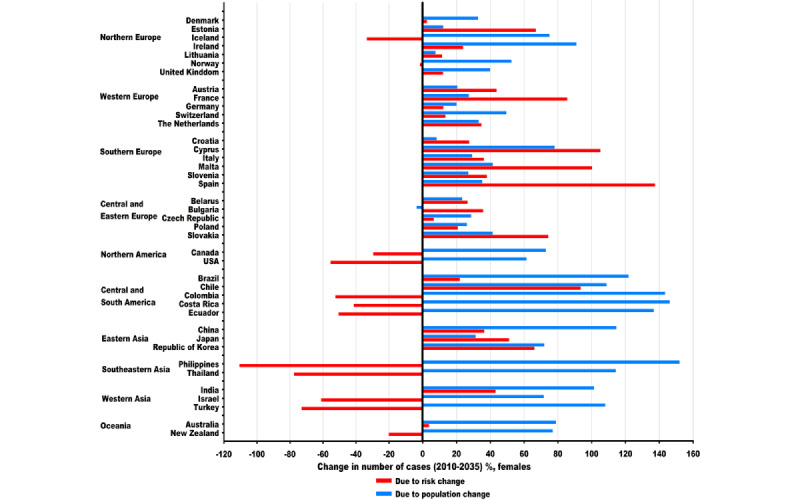
Projected changes in the number of new lung cancer cases between 2010 and 2035 in 40 countries among females, partitioned into population and risk change. Numbers for 2010 and 2035 represent average numbers for the 5-year period centered on the respective year. Due to population change: projected change in the number of cases due to population growth and aging. Due to risk change: projected change in the number of cases due to changes in incidence rates. Blue color stands for population change, and red color stands for risk change.

When analyzed by country, the number of new cases is expected to increase in 32 countries among males and in all 40 countries among females between 2010 and 2035 (Multimedia Appendices 2 and 3). The predicted increases among males in most of these 32 countries are mainly due to population growth and aging, regardless of marked decreases in LC risk, whereas those among females in most countries in Southern, Western, and Central and Eastern Europe are largely due to marked increases in LC risk (Figures 6 and 7). The most rapid increases were predicted in Cyprus (459/257, 178.3%) among males and Chile (1253/620, 202.2%) among females (Multimedia Appendices 2 and 3). In 2035, the largest number of new cases was projected in China (655,000), Japan (112,000), and the United States (105,000) among males and China (451,000), the United States (95,000), and Japan (61,000) among females.

### Cumulative Risk of LC Incidence in 2035

**Figure 8:**
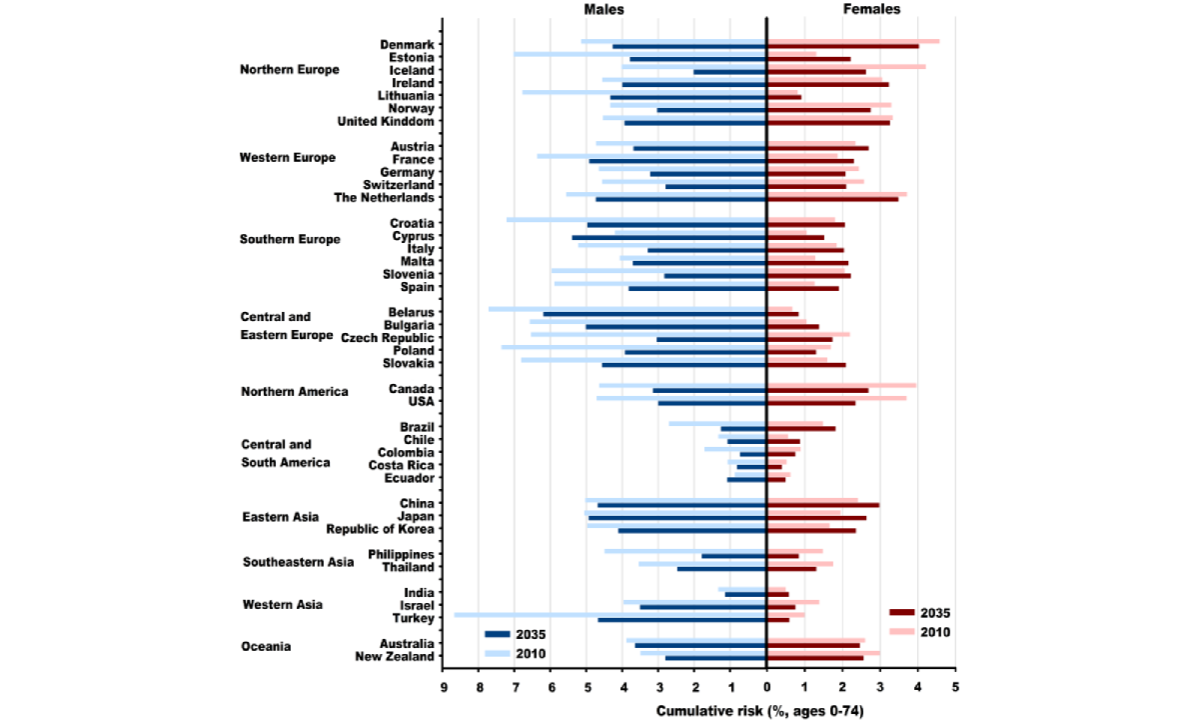
Cumulative risk (in percent, ages 0-74 years) of lung cancer in 2010 and 2035 in 40 countries. Blue color stands for males, and red color stands for females.

The cumulative risks among males are expected to decrease in almost all countries (except Cyprus and Ecuador) between 2010 and 2035, whereas those among females are expected to increase in 20 countries (especially in Eastern Asian and Southern European countries; Figure 8; Multimedia Appendices 2 and 3). In 2035, the highest cumulative risk among males is expected in Belarus (6.2/100, 6.2%), Cyprus (5.4/100, 5.4%), Bulgaria (5/100, 5%), Croatia (5/100, 5%), and Japan (4.9/100, 4.9%), which indicates that approximately 5 to 6 of 100 males will be diagnosed with LC before the age of 75 years in the aforementioned countries. Among females, the highest cumulative risks are expected in Denmark (4/100, 4%), the Netherlands (3.5/100, 3.5%), the United Kingdom (3.3/100, 3.3%), Ireland (3.2/100, 3.2%), and China (3/100, 3%).

## Discussion

### Principal Findings

In our study, the ASRs of LC among females are predicted to increase in half of the countries between 2010 and 2035, while those among males are expected to decrease in almost all studied countries. The ASRs among females are expected to peak after the 2020s in most European, Eastern Asian, and Oceanian countries and will reach and surpass those among males in 11 countries after the 2020s. In 2035, high rates are predicted to mainly occur among males in Central and Eastern European countries and among females in Northern European countries. The largest increases in the number of new cases in both sexes between 2010 and 2035 are predicted in Eastern Asia. China will have the largest number of new cases in both sexes by 2035.

### Interpretation of Changes in LC Incidence Rates

Tobacco smoking is the leading risk factor for LC, with 76.2% (74.6 to 77.8) of LC-related deaths among males and 38.9% (36.7 to 40.9) of LC-related deaths among females attributed to smoking in 2019 [3]. Temporal trends in LC incidence are largely affected by smoking patterns in the past 30 to 40 years [23,24], and the future LC burden will depend on historic smoking patterns at the population level [25]. Smoking prevalence among males in most European countries (eg, the United Kingdom and Spain) peaked during the 1950s and 1960s, whereas that among females peaked in the 1980s and 1990s [23,26-32]. For example, the smoking prevalence among Spanish females increased from 5.8% in 1970 to 26.5% in 1990 and then decreased after the 2000s [23,30]; this could largely explain the predicted increase in the LC incidence rate and the peak LC rate in 2025 among females. Similarly, the lag duration of 30 to 40 years combined with the maximum smoking prevalence during the 1980s and 1990s [26,27,31,32] could indicate that LC incidence rates among females will peak after the 2020s in many European countries. In addition, a recent study showed that the smoking-attributable fractions of LC mortality among males are predicted to decline in Denmark and the Netherlands from 2009 to 2050, whereas those among females will increase until it peaks in 2028 in Denmark and 2033 in the Netherlands [33], similar to our projections of changes in LC rates from 2010 to 2035. Notably, smoking patterns in younger birth cohorts may directly indicate future LC incidence rates [32,34,35]. The predicted increase in LC incidence rates among females in most European countries could be largely explained by the cohort effect because of the increased smoking prevalence in cohorts born after the 1950s [32,36]. The predicted decreases in LC incidence rates in the United States in both sexes might largely result from changes in smoking habits in cohorts born after the 1940s, who mainly smoked low-tar and filter-tipped cigarettes [2,37,38].

### Interpretation of the Discrepancy of Changes in LC Incidence Rates Between Sexes

The convergence of decreasing male and increasing female incidence rates of LC in our study could be largely explained by the changes in smoking prevalence. Globally, females started smoking later than males (the 1950s), but the smoking prevalence increased rapidly until the 1990s and then remained relatively stable [23,26-32,39]. For example, in France, tobacco smoking increased from 0.4 daily smoked cigarettes per woman in 1953 to 3.7 in 1991 and thereafter remained stable, whereas males smoked approximately 8.6 cigarettes per day in 1953 until a peak in 1980 (9.6 cigarettes per day) and declined steadily (5.2 cigarettes per day in 2003) [39]. Notably, smoking behavior and smoking prevalence among males and females have become increasingly similar in the younger generation worldwide [40,41], indicating the convergence of decreasing male and increasing female incidence rates of LC.

### Interpretation of Geographic Distributions in Predicted LC Incidence Rates and Cumulative Risks

In 2035, the incidence rates of LC are projected to vary significantly across regions for both sexes. The highest predicted rate and cumulative risk among males in Central and Eastern European countries could be largely attributable to the historically higher smoking prevalence than that of Western countries and delays in the implementation of smoking prevention and cessation measures [42-44]. LC mortality rates attributable to smoking were also estimated to be the highest in Central and Eastern European countries in 2019 [3]. Among females, the highest predicted LC rate and cumulative risk in Northern European countries could be largely explained by the historically high LC rate and the fact that smoking prevalence among females only began to decrease after the late 1980s [31,32].

### Interpretation of Changes in New LC Cases

Worldwide, the reduction in LC risk among males will only partially offset the expected increase in the number of new LC cases because of population growth and aging. However, the increasing future LC burden among females will be driven by increasing LC risks, and thus mainly tobacco smoking patterns and trends, in most countries in Southern, Western, and Central and Eastern Europe. In addition, the largest number of new LC cases is predicted in China, accounting for nearly half (45%) of the total number of new cases among all studied countries in 2035, and more than half (60%) of the total increase in the number of new cases between 2010 and 2035. A recent study showed that the LC mortality rates attributed to smoking and ambient particulate matter pollution in China were estimated to be 80.8% and 20.8% among males and 23.4% and 19.7% among females in 2017, respectively [45]. China has the largest smoking population in the world, with an estimated 301 million smokers, and the smoking proportions of males and females in the Chinese population are 52.9% and 2.4% in 2010 [46]. More than half of the LC cases attributed to exposure to ambient particulate matter pollution have been reported in China and other East Asian countries [47,48]. In addition, China has experienced rapid population growth and a transition to an aging society in recent decades [49]. Thus, the substantial future burden of LC in China might largely result from the large smoking population, deteriorating air pollution, and changing demographics, and will, to some extent, hinder the realization of global noncommunicable disease control goals with a reduction of one-third in premature mortality from noncommunicable diseases by 2030 [50]. Tobacco control programs and clean air legislation should be strengthened and additional resources should be allocated to reduce the LC burden in China.

### Comparison With Prior Work

The Global Cancer Observatory of the International Agency for Research on Cancer provides the future LC incidence worldwide by 2040, based on demographic projections estimated by the United Nations Development Programme and scenarios of stable rates from the baseline year 2020 [51]. However, projections of future LC incidence by 2040 in the Global Cancer Observatory did not consider changes in the incidence rates of LC. Our predictions have accounted for historical changes in LC incidence rates using the NORDPRED age-period-cohort model, which could largely reflect the real situation of LC incidence. To the best of our knowledge, this is the first study to predict the future burden of LC incidence from a global perspective based on incidence data from high-quality population-based cancer registries, enabling direct comparisons of findings between countries. The well-known modern method based on the NORDPRED age-period-cohort model was applied to provide estimates of future LC burden in our study.

### Limitations

However, this study has some limitations. First, the trend-based predictions in our study might have a certain degree of uncertainty because we assumed that the historical trends in LC incidence will continue into the future in the model [5]. However, when comparing our predicted ASRs with those already observed in, for example, Australia, Norway, and the Netherlands, these figures seem fairly close (eg, observed ASR 30.7 in Australian males in 2015 [52] vs 30.9 predicted in 2015, 21.8 in Australian females [52] vs 22.4; observed ASR 31.3 in Norwegian males in 2015 [53] vs 33.6 predicted in 2015, 28.0 in Norwegian females [53] vs 27.6; and observed ASR 38.6 in Dutch males in 2019 [54] vs 41.4 predicted in 2020, 33.1 in Dutch females [54] vs 35.7); these might largely indicate the accuracy of our projections. Second, for lower-risk and small populations (eg, Malta and Cyprus), random variation might exist. Third, we were unable to predict the future LC burden in many countries in Africa, Latin America, and Asia because of the lack of high-quality data. Fourth, the aggregated data from multiple regional registries in a given country might not fully represent the national level, although this method has been applied to cover the largest geographic area in many previously published papers [2,55,56]. Figures from regional cancer registries with limited resources could be underestimated and those from registries with abundant resources could be overestimated. Caution should be exercised when interpreting these findings.

### Conclusions

The LC incidence rates among females are predicted to continue increasing dramatically in most countries by 2035, with peaks after the 2020s in most European, Eastern Asian, and Oceanian countries, whereas incidence rates among males are predicted to continue declining in almost all countries. The sex difference in the worldwide incidence of LC will further narrow down in the future. Geographic variations in LC incidence rates will remain high, with the highest rate in Central and Eastern Europe among males and in Northern Europe among females. The number of new LC cases are expected to continue to increase through 2035 in most countries because of population aging and growth, as well as changes in the prevalence of risk factors, making LC a major challenge to public health worldwide. These ongoing transitions in LC epidemiology are highly relevant to future cancer control and clinical practice. Clinicians can expect to observe more cases of LC in the future. The findings highlight the need for the redistribution of resources and improved LC control measures (eg, smoking prevention and cessation programs and air quality management programs) to reduce the future LC burden worldwide.

## Appendix

Multimedia Appendix 1 Statistical analysis of the assessment of the prediction performance of the NORDPRED age-period-cohort model and the corresponding findings.

Multimedia Appendix 2 Number of new lung cancer cases and age-standardized incidence rates in lung cancer incidence in 40 countries in 2010 and 2035 among males and the corresponding percentage change between 2010 and 2035.

Multimedia Appendix 3 Number of new lung cancer cases and age-standardized incidence rates in lung cancer incidence in 40 countries in 2010 and 2035 among females and the corresponding percentage change between 2010 and 2035.

## Abbreviations

ASR: age-standardized incidence rate
LC: lung cancer

## Data Availability

The Cancer Incidence in Five Continents data are publicly available on the web [7]. The processed data sets used in the analysis are available from the corresponding author upon reasonable request.
