# Supplementary material for: Projections of Lung Cancer Incidence by 2035 in 40 Countries Worldwide: Population-Based Study
Source: JMIR Public Health Surveill. 2023 Feb 17;9:e43651. doi: 10.2196/43651 (PMC9984998; doi:10.2196/43651)
Supplement: Multimedia Appendix 1 [file publichealth_v9i1e43651_app1.docx]

## Multimedia Appendix 1

## Methods

### Statistical Analysis

We assessed the prediction performance of the NORDPRED age-period-cohort model by splitting LC incidence data into a training dataset and a verification dataset. Here, given the data demand for verification, we can only examine the predictive performance of the model in countries with at least three calendar-year periods. The data of the previous three calendar-year periods were used for the training dataset, and the data of the remaining calendar periods (depending on the available data) were used for the verification dataset. For example, in Denmark, the training dataset was from 1978-1992, and the verification dataset was from 1993-2012; in Ireland, the training dataset was from 1993-2007, and the verification dataset was from 2008-2012. The root-mean-squared error (RMSE) and the error rate (ER) were used to assess the predictive performance of the NORDPRED age-period-cohort model. This validation method has been performed in many previously published papers [1-4]. The RMSE was calculated as follows:

$$RMSE=\sqrt{\frac{1}{n}\sum_{i=1}^{n} {(\hat{y}_{i}-y_{i})}^{2}}$$

where $i$ is the calendar period in the verification dataset, and $\hat{y}_{i}$ and $y_{i}$ denote the prediction rates and the observational rates, respectively [1]. The RMSE is measured in the original scale of the rate, and assess the performance of the model in each country. The RMSE is always non-negative, and a value close to 0 indicates a good performance of the model. Therefore, a lower RMSE is better than a higher one. However, comparisons across different regions would be invalid because the measure is dependent on the scale of the rate. Usually, countries with the highest rates provide highest RMSE value. Thus, the interpretation of the RMSE should be accompanied by the rate.

The ER was calculated as follows:

$$ER=\frac{1}{n}\sum_{i=1}^{n} \frac{\left| \hat{y}_{i}-y_{i} \right|}{y_{i}}$$

where $i$ is the calendar period in the verification dataset, and $\hat{y}_{i}$ and $y_{i}$ denote the prediction rates and the observational rates, respectively [5]. The ER is a relative measurement independent on the scale of the rate allowing the comparison between countries. The lower ER indicates the better prediction performance. A global ER was also computed, as the mean of the country-specific ER for the global performance of the model.

Mean annual differences in the numbers of predicted cases in 2035 (the midpoint of calendar period 2033-2037) relative to 2010 (the midpoint of calendar period 2008-2012) are partitioned into changes in risk (rates) and changes in demographics (population size and age structure) [6]. Let $N_{ras}$ be the number of cases given a cancer risk $r$, an age structure $a$ and a population size $s$, where $r$, $a$ and $s$ are levels for the observed year 2010 ($o$) or the future year 2035 ($f$). $N_{000}$ is then the expected number of cases applying the rates, age structure and population size of 2010, which is the observed number of cases in 2010. Similarly, $N_{fff}$ is the predicted number of cases in 2035. $N_{fff}$ – $N_{000}$ is the change in the annual number of cases, and this increase (or decrease) can be decomposed into two components:

$\Delta_{tot}$ = $N_{fff}$ – $N_{000}$ = ($N_{fff}$ – $N_{off}$) + ($N_{off}$ – $N_{000}$) = $\Delta risk+\Delta pop,$

where $N_{off}$ is the expected number of cases in 2035 when the rates in 2010 are applied. The component $\Delta risk$ is thus the difference between the numbers of cases in the future calculated using the predicted or observed rates, while $\Delta pop$ is the difference between the number of cases found when present rates are applied to present or future age distribution and population size. The two components can differ from zero in either a positive or a negative direction.

## Results

### The Prediction Performance of the NORDPRED Age-period-cohort Model

The prediction performance of the NORDPRED age-period-cohort model by country and gender is presented in Table 1. The global ER was 0.187 for males and 0.183 for females. The vast majority of countries showed the low RMSE and ER. These figures indicate the good prediction performance of the NORDPRED age-period-cohort model.

| Table 1. The prediction performance of the NORDPRED age-period-cohort model by country | | | | | |
| --- | --- | --- | --- | --- | --- |
| Country | Male | |  | Female | |
|  | RMSE^a^ | ER^b^ |  | RMSE^a^ | ER^b^ |
| Northern Europe | | | | | |
| Denmark | 5.362 | 0.115 |  | 7.890 | 0.204 |
| Estonia | 4.514 | 0.059 |  | 1.589 | 0.104 |
| Iceland | 3.645 | 0.095 |  | 7.988 | 0.267 |
| Ireland | 1.518 | 0.039 |  | 0.888 | 0.035 |
| Lithuania | 1.091 | 0.020 |  | 0.236 | 0.031 |
| Norway | 4.278 | 0.107 |  | 5.251 | 0.185 |
| United Kingdom | 15.674 | 0.355 |  | 8.242 | 0.311 |
| Western Europe | | | | | |
| Austria | Not available^c^ | Not available^c^ |  | Not available^c^ | Not available^c^ |
| France | 1.423 | 0.023 |  | 4.364 | 0.257 |
| Germany | 22.356 | 0.480 |  | 4.024 | 0.207 |
| Switzerland | 15.828 | 0.356 |  | 4.408 | 0.216 |
| The Netherlands | 4.219 | 0.086 |  | 2.962 | 0.101 |
| Southern Europe | | | | | |
| Croatia | 5.064 | 0.081 |  | 0.254 | 0.013 |
| Cyprus | Not available^c^ | Not available^c^ |  | Not available^c^ | Not available^c^ |
| Italy | 25.519 | 0.470 |  | 6.877 | 0.468 |
| Malta | 6.327 | 0.179 |  | 3.210 | 0.294 |
| Slovenia | 12.131 | 0.218 |  | 2.999 | 0.183 |
| Spain | 19.293 | 0.357 |  | 3.997 | 0.409 |
| Central and Eastern Europe | | | | | |
| Belarus | 6.022 | 0.096 |  | 1.900 | 0.326 |
| Bulgaria | Not available^c^ | Not available^c^ |  | Not available^c^ | Not available^c^ |
| Czech Republic | 6.350 | 0.105 |  | 0.674 | 0.042 |
| Poland | Not available^c^ | Not available^c^ |  | Not available^c^ | Not available^c^ |
| Slovakia | 11.503 | 0.188 |  | 1.877 | 0.159 |
| Northern America | | | | | |
| Canada | 1.769 | 0.040 |  | 1.780 | 0.053 |
| USA | 5.781 | 0.119 |  | 1.812 | 0.053 |
| Central and South America | | | | | |
| Brazil | 9.910 | 0.466 |  | 2.546 | 0.210 |
| Chile | Not available^c^ | Not available^c^ |  | Not available^c^ | Not available^c^ |
| Colombia | 1.672 | 0.093 |  | 0.527 | 0.058 |
| Costa Rica | 1.769 | 0.170 |  | 0.931 | 0.188 |
| Ecuador | 0.745 | 0.076 |  | 0.773 | 0.148 |
| Eastern Asia | | | | | |
| China | 1.674 | 0.032 |  | 2.154 | 0.102 |
| Japan | 1.224 | 0.024 |  | 2.043 | 0.100 |
| Republic of Korea | 1.463 | 0.034 |  | 1.007 | 0.069 |
| Southeastern Asia | | | | | |
| Philippines | 6.226 | 0.138 |  | 0.744 | 0.040 |
| Thailand | 3.880 | 0.103 |  | 4.516 | 0.280 |
| Western Asia | | | | | |
| India | 15.930 | 1.429 |  | 2.934 | 0.735 |
| Israel | 1.133 | 0.033 |  | 2.305 | 0.172 |
| Turkey | Not available^c^ | Not available^c^ |  | Not available^c^ | Not available^c^ |
| Oceania | | | | | |
| Australia | 1.476 | 0.038 |  | 3.370 | 0.115 |
| New Zealand | 4.584 | 0.132 |  | 2.074 | 0.086 |
| ^a^ RMSE: root-mean-squared error.  ^b^ ER: error rate.  ^c^ the LC incidence data were not available for the assessment of the prediction performance. | | | | | |

## References

1. Yu J, Yang X, He W, Ye W. Burden of pancreatic cancer along with attributable risk factors in Europe between 1990 and 2019, and projections until 2039. International journal of cancer. 2021 Sep 1;149(5):993-1001. PMID: 33937984. doi: 10.1002/ijc.33617.

2. Liu Z, Xu K, Jiang Y, Cai N, Fan J, Mao X, et al. Global trend of aetiology-based primary liver cancer incidence from 1990 to 2030: a modelling study. International journal of epidemiology. 2021 Mar 3;50(1):128-42. PMID: 33349860. doi: 10.1093/ije/dyaa196.

3. Cheng F, Xiao J, Shao C, Huang F, Wang L, Ju Y, et al. Burden of Thyroid Cancer From 1990 to 2019 and Projections of Incidence and Mortality Until 2039 in China: Findings From Global Burden of Disease Study. Frontiers in endocrinology. 2021;12:738213. PMID: 34690931. doi: 10.3389/fendo.2021.738213.

4. Du Z, Chen W, Xia Q, Shi O, Chen Q. Trends and projections of kidney cancer incidence at the global and national levels, 1990-2030: a Bayesian age-period-cohort modeling study. Biomarker research. 2020;8:16. PMID: 32435498. doi: 10.1186/s40364-020-00195-3.

5. Lee TC, Dean CB, Semenciw R. Short-term cancer mortality projections: a comparative study of prediction methods. Statistics in medicine. 2011 Dec 20;30(29):3387-402. PMID: 21965149. doi: 10.1002/sim.4373.

6. Møller B, Fekjaer H, Hakulinen T, Tryggvadóttir L, Storm HH, Talbäck M, et al. Prediction of cancer incidence in the Nordic countries up to the year 2020. European journal of cancer prevention : the official journal of the European Cancer Prevention Organisation (ECP). 2002 Jun;11 Suppl 1:S1-96. PMID: 12442806.
