# Supplementary material for: Projections of Lung Cancer Incidence by 2035 in 40 Countries Worldwide: Population-Based Study
Source: JMIR Public Health Surveill. 2023 Feb 17;9:e43651. doi: 10.2196/43651 (PMC9984998; doi:10.2196/43651)
Supplement: Multimedia Appendix 3 [file publichealth_v9i1e43651_app3.docx]

**Multimedia Appendix 3.** Number of new lung cancer cases and age-standardized incidence rates in lung cancer incidence in 40 countries in 2010 and 2035 in women and the corresponding percentage change between 2010 and 2035.

| Country | | Population (annual; million) | | Number of new cases (annual) | | Change in incidence (%) | Change in incidence due to population (%) | Change in incidence due to risk (%) | ASR^a^ | | ASR rank | | ASR annual change (%) | ASR change (%) | Cumulative risk  (%; 0-74 years) | |
| --- | --- | --- | --- | --- | --- | --- | --- | --- | --- | --- | --- | --- | --- | --- | --- | --- |
|  | | 2010 | 2035 | 2010 | 2035 |  |  |  | 2010 | 2035 | 2010 | 2035 |  |  | 2010 | 2035 |
|  | | | | | | | | | | | | | | | | |
| Overall | | 2012.2 | 2311.7 | 459,606 | 889,074 | 93.4 | — | — | — | — | — | — | — | — | — | — |
| **Northern Europe** | | 42.4 | 46.9 | 24,729 | 37,708 | 52.5 | — | — | — | — | — | — | — | — | — | — |
|  | Denmark | 2.8 | 3.1 | 2154 | 2909 | 35.0 | 32.7 | 2.4 | 36.8 | 32.9 | 1 | 1 | -2.3 | -10.4 | 4.57 | 4.03 |
|  | Estonia | 0.7 | 0.6 | 181 | 325 | 79.1 | 12.1 | 66.8 | 10.7 | 18.0 | 32 | 20 | 9.7 | 67.8 | 1.30 | 2.21 |
|  | Iceland | 0.2 | 0.2 | 85 | 121 | 41.6 | 74.9 | -33.3 | 33.0 | 23.8 | 3 | 6 | -6.7 | -28.0 | 4.20 | 2.62 |
|  | Ireland | 2.3 | 2.7 | 920 | 1975 | 114.7 | 90.9 | 23.8 | 25.4 | 27.1 | 10 | 4 | 1.0 | 6.8 | 3.05 | 3.23 |
|  | Lithuania | 1.7 | 1.3 | 287 | 340 | 18.8 | 7.4 | 11.4 | 7.3 | 7.8 | 37 | 33 | 1.4 | 7.2 | 0.81 | 0.91 |
|  | Norway | 2.4 | 3.0 | 1206 | 1822 | 51.1 | 52.4 | -1.2 | 25.7 | 22.8 | 9 | 9 | -2.7 | -11.2 | 3.29 | 2.75 |
|  | United Kingdom^b^ | 32.3 | 36.0 | 19,897 | 30,215 | 51.9 | 39.8 | 12.0 | 27.3 | 28.0 | 8 | 3 | 0.2 | 2.6 | 3.33 | 3.25 |
| **Western Europe** | | 90.3 | 94.4 | 35,736 | 57,566 | 61.1 | — | — | — | — | — | — | — | — | — | — |
|  | Austria | 4.3 | 4.6 | 1620 | 2658 | 64.1 | 20.4 | 43.7 | 19.0 | 22.2 | 16 | 11 | 2.8 | 17.2 | 2.34 | 2.69 |
|  | France^b^ | 32.4 | 34.7 | 9562 | 20,334 | 112.7 | 27.2 | 85.4 | 15.8 | 19.1 | 20 | 18 | 3.2 | 20.3 | 1.87 | 2.29 |
|  | Germany^b^ | 41.3 | 41.5 | 18,334 | 24,225 | 32.1 | 20.0 | 12.2 | 19.6 | 17.8 | 15 | 22 | -2.3 | -9.5 | 2.43 | 2.08 |
|  | Switzerland^b^ | 4.0 | 4.7 | 1625 | 2644 | 62.7 | 49.4 | 13.3 | 20.4 | 18.0 | 14 | 19 | -2.8 | -12.1 | 2.57 | 2.10 |
|  | The Netherlands | 8.4 | 8.8 | 4595 | 7705 | 67.6 | 33.0 | 34.6 | 29.5 | 28.7 | 7 | 2 | -1.0 | -2.9 | 3.71 | 3.47 |
| **Southern Europe** | | 58.3 | 56.7 | 17,528 | 33,772 | 92.7 | — | — | — | — | — | — | — | — | — | — |
|  | Croatia | 2.2 | 1.9 | 738 | 1000 | 35.6 | 8.2 | 27.4 | 15.2 | 16.8 | 22 | 25 | 1.9 | 10.5 | 1.79 | 2.06 |
|  | Cyprus | 0.6 | 0.7 | 75 | 214 | 183.1 | 78.0 | 105.1 | 8.7 | 13.5 | 34 | 29 | 8.0 | 54.0 | 1.05 | 1.51 |
|  | Italy^b^ | 30.6 | 29.6 | 11,741 | 19,404 | 65.3 | 29.2 | 36.1 | 15.4 | 17.6 | 21 | 23 | 2.3 | 13.7 | 1.84 | 2.04 |
|  | Malta | 0.2 | 0.2 | 47 | 112 | 141.6 | 41.5 | 100.1 | 10.9 | 17.4 | 30 | 24 | 9.5 | 59.8 | 1.27 | 2.15 |
|  | Slovenia | 1.0 | 1.0 | 361 | 595 | 64.9 | 26.9 | 37.9 | 17.0 | 19.1 | 18 | 17 | 2.0 | 12.4 | 2.05 | 2.21 |
|  | Spain^b^ | 23.7 | 23.3 | 4567 | 12,448 | 172.6 | 35.2 | 137.4 | 10.8 | 14.9 | 31 | 28 | 5.9 | 37.8 | 1.25 | 1.89 |
| **Central and Eastern Europe** | | 36.8 | 34.8 | 8780 | 13,036 | 48.5 | — | — | — | — | — | — | — | — | — | — |
|  | Belarus | 5.0 | 4.8 | 565 | 846 | 49.8 | 23.3 | 26.5 | 5.5 | 6.7 | 39 | 35 | 3.7 | 21.0 | 0.66 | 0.83 |
|  | Bulgaria | 3.8 | 3.2 | 681 | 903 | 32.5 | -3.2 | 35.7 | 8.7 | 11.1 | 33 | 30 | 4.7 | 27.2 | 1.04 | 1.38 |
|  | Czech Republic | 5.4 | 5.4 | 1967 | 2657 | 35.1 | 28.6 | 6.4 | 17.3 | 15.1 | 17 | 27 | -3.0 | -13.0 | 2.20 | 1.73 |
|  | Poland^b^ | 19.8 | 18.6 | 4903 | 7199 | 46.8 | 26.1 | 20.7 | 13.4 | 10.7 | 25 | 31 | -4.3 | -19.9 | 1.69 | 1.30 |
|  | Slovakia | 2.8 | 2.7 | 664 | 1431 | 115.6 | 41.4 | 74.3 | 13.1 | 17.9 | 26 | 21 | 5.5 | 37.0 | 1.59 | 2.09 |
| **Northern America** | | 173.5 | 202.4 | 100,077 | 110,076 | 10.0 |  |  |  |  |  |  |  |  |  |  |
|  | Canada^b^ | 17.2 | 21.2 | 10,646 | 15,244 | 43.2 | 72.8 | -29.6 | 31.5 | 22.6 | 4 | 10 | -6.4 | -28.1 | 3.95 | 2.68 |
|  | United States^b^ | 156.3 | 181.1 | 89,431 | 94,832 | 6.0 | 61.4 | -55.4 | 30.2 | 19.6 | 6 | 16 | -8.4 | -35.2 | 3.69 | 2.34 |
| **Central and South America** | | 140.6 | 167.1 | 16,226 | 38,566 | 137.7 | — | — | — | — | — | — | — | — | — | — |
|  | Brazil^b^ | 99.2 | 116.1 | 13,105 | 31,924 | 143.6 | 121.8 | 21.8 | 12.1 | 15.2 | 28 | 26 | 4.9 | 25.7 | 1.47 | 1.81 |
|  | Chile^b^ | 8.7 | 10.0 | 620 | 1872 | 202.2 | 108.7 | 93.4 | 4.7 | 6.3 | 40 | 38 | 7.7 | 33.3 | 0.55 | 0.86 |
|  | Colombia^b^ | 23.0 | 27.8 | 1966 | 3752 | 90.9 | 143.3 | -52.4 | 8.0 | 6.5 | 36 | 36 | -4.1 | -18.2 | 0.89 | 0.75 |
|  | Costa Rica | 2.3 | 2.8 | 110 | 226 | 104.7 | 146.1 | -41.3 | 4.3 | 3.3 | 41 | 42 | -5.1 | -23.5 | 0.51 | 0.38 |
|  | Ecuador^b^ | 7.5 | 10.4 | 425 | 792 | 86.1 | 136.6 | -50.5 | 5.9 | 4.8 | 38 | 41 | -4.3 | -19.3 | 0.61 | 0.49 |
| **Eastern Asia** | | 743.7 | 789.9 | 216,161 | 518,886 | 140.0 |  |  |  |  |  |  |  |  |  |  |
|  | China^b^ | 665.5 | 716.0 | 179,915 | 451,385 | 150.9 | 114.5 | 36.4 | 20.8 | 24.7 | 13 | 5 | 3.6 | 18.7 | 2.41 | 2.97 |
|  | Japan^b^ | 65.6 | 60.1 | 33,683 | 61,401 | 82.3 | 31.2 | 51.1 | 16.8 | 23.3 | 19 | 7 | 6.2 | 38.4 | 1.94 | 2.63 |
|  | Republic of Korea^b^ | 12.6 | 13.7 | 2563 | 6099 | 137.9 | 71.8 | 66.1 | 14.7 | 19.9 | 23 | 15 | 6.1 | 35.8 | 1.65 | 2.36 |
| **Southeastern Asia** | | 80.9 | 101.3 | 10,745 | 14,876 | 38.5 | — | — | — | — | — | — | — | — | — | — |
|  | Philippines^b^ | 46.6 | 65.1 | 4210 | 5946 | 41.2 | 151.8 | -110.6 | 12.2 | 6.9 | 27 | 34 | -11.1 | -43.6 | 1.47 | 0.84 |
|  | Thailand^b^ | 34.3 | 36.1 | 6534 | 8931 | 36.7 | 114.1 | -77.4 | 14.0 | 10.6 | 24 | 32 | -5.6 | -24.3 | 1.74 | 1.30 |
| **Western Asia** | | 632.4 | 800.8 | 24,431 | 55,355 | 126.6 |  |  |  |  |  |  |  |  |  |  |
|  | India^b^ | 591.9 | 748.9 | 20,612 | 50,353 | 144.3 | 101.3 | 43.0 | 4.1 | 4.9 | 42 | 39 | 3.2 | 18.9 | 0.49 | 0.58 |
|  | Israel | 3.7 | 5.3 | 625 | 691 | 10.5 | 71.5 | -61.0 | 11.4 | 6.4 | 29 | 37 | -10.7 | -43.4 | 1.38 | 0.75 |
|  | Turkey^b^ | 36.8 | 46.5 | 3194 | 4311 | 35.0 | 107.9 | -72.9 | 8.1 | 4.8 | 35 | 40 | -9.6 | -40.2 | 0.98 | 0.59 |
| **Oceania** | | 13.3 | 17.5 | 5192 | 9232 | 77.8 | — | — | — | — | — | — | — | — | — | — |
|  | Australia^b^ | 11.1 | 14.8 | 4256 | 7764 | 82.4 | 78.7 | 3.7 | 21.0 | 20.9 | 12 | 12 | -0.4 | -0.5 | 2.60 | 2.45 |
|  | New Zealand | 2.2 | 2.7 | 936 | 1468 | 56.7 | 76.8 | -20.1 | 24.2 | 20.8 | 11 | 13 | -3.1 | -14.1 | 2.98 | 2.56 |

^a^ASR: age-standardized incidence rates.

^b^Regional registries: United Kingdom (East England, East Midlands, London, North East, North West, South East, South West, West Midlands, Yorkshire-Humber, Northern Ireland, and Scotland), France (Bas-Rhin, Calvados, Doubs, Haut-Rhin, Herault, Isere, Loire-Atlantique, Manche, Somme, and Tarn), Germany (Hamburg, Saarland, and Munich), Switzerland (Geneva, Neuchatel, St Gall-Appenzell, Ticino, Valais, Vaud, and Graubunden and Glarus), Italy (Biella, Ferrara, Modena, Naples, Parma, Ragusa, Romagna, Veneto, Reggio Emilia, and Sondrio), Spain (Albacete, Basque Country, Canary Islands, Cuenca, Girona, Granada, Murcia, Navarra, and Tarragona), Poland (Kielce), Canada (Alberta, British Columbia, Manitoba, New Brunswick, Newfoundland and Labrador, Northwest Territories, Nova Scotia, Ontario, Prince Edward Island, and Saskatchewan), United States (SEER9 that includes the states of Connecticut, Hawaii, Iowa, New Mexico, and Utah, and the areas of Detroit in Michigan, San Francisco in California, Atlanta in Georgia, and Seattle in Washington), Brazil (Goiana), Chile (Valdivia), Colombia (Cali), Ecuador (Quito), China (Hong Kong, Jiashan, Shanghai, Harbin, and Zhongshan), Japan (Miyagi, Nagasaki, Osaka, Fukui, and Aichi), Republic of Korea (Busan, Gwangju, Incheon, Seoul, Ulsan, and Daejeon), Philippines (Manila), Thailand (Chiang Mai, KhonKaen, Lampang, and Songkhla), India (Chennai and Mumbai), Turkey (Antalya and Izmir), and Australia (New South Wales, Australian Capital Territory, Queensland, South Australia, Tasmania, Victoria, Western Australia, and Northern Territories).

The em dashes in empty cells indicate not applicable.
